# Rational Design of Low-Band Gap Star-Shaped Molecules With 2,4,6-Triphenyl-1,3,5-triazine as Core and Diketopyrrolopyrrole Derivatives as Arms for Organic Solar Cells Applications

**DOI:** 10.3389/fchem.2019.00122

**Published:** 2019-03-19

**Authors:** Xinhao Zhang, Ruifa Jin

**Affiliations:** ^1^Inner Mongolia Key Laboratory of Photoelectric Functional Materials, Chifeng University, Chifeng, China; ^2^College of Chemistry and Chemical Engineering, Chifeng University, Chifeng, China

**Keywords:** star-shaped molecules, diketopyrrolopyrrole derivatives, optical and electronic properties, frontier molecular orbitals (FMOs), organic solar cells (OSCs)

## Abstract

A series of D–A novel star-shaped molecules with 2,4,6-triphenyl-1,3,5-triazine (TPTA) as core, diketopyrrolo[3,4-c]pyrrole (DPP) derivatives as arms, and triphenylamine (TPA) derivatives as end groups have been systematically investigated for organic solar cells (OSCs) applications. The electronic, optical, and charge transport properties were studied using density functional theory (DFT) and time-dependent DFT (TD-DFT) approaches. The parameters such as energetic driving force Δ*E*_L−L_, adiabatic ionization potential *AIP*, and adiabatic electron affinity *AEA* were also calculated at the same level. The calculated results show that the introduction of different groups to the side of DPP backbones in the star-shaped molecules can tune the frontier molecular orbitals (FMOs) energy of the designed molecules. The designed molecules can provide match well with those of typical acceptors PCBM ([6,6]-phenyl-C61-butyric acid methyl ester) and PC71BM ([6,6]-phenyl-C71-butyric acid methyl ester). Additionally, the absorption wavelengths of the designed molecules show bathochromic shifts compared with that of the original molecule, respectively. The introduction of different groups can extend the absorption spectrum toward longer wavelengths, which is beneficial to harvest more sunlight. The calculated reorganization energies suggest that the designed molecules are expected to be the promising candidates for ambipolar charge transport materials except molecule with benzo[c]isothiazole group can be used as hole and electron transport material. Moreover, the different substituent groups do not significantly affect the stability of the designed molecules.

## Introduction

Nowadays, organic π-conjugated small molecules (SMs) used as the donors in organic solar cells (OSCs) have drawn intense attention because of their outstanding advantages, such as excellent reproducibility, easy purification, well-defined chemical and optoelectronic properties (Coughlin et al., 2014; Yao et al., 2016; Bin et al., 2017). Owing to the tremendous efforts on improving the performance of OSCs based on SMs, their power conversion efficiency (PCE) has surpassed over 10% recently (Zhou et al., 2012; Kan et al., 2015). However, it is worth noting that their overall performance still falls behind that of their polymer counterparts (Ni et al., 2013; Lin and Zhan, 2016). Accordingly, to address this issue, it is a big challenge to design and synthesize high-performance and desirable donor novel SMs (Chaudhry et al., 2018; Irfan et al., 2018; Wazzan et al., 2018). In general, the high-efficiency SMs donor materials should possess suitable frontier molecular orbital (FMOs) (including the highest occupied molecular orbital, HOMO, and lowest unoccupied molecular orbital, LUMO) energy levels, high charge carrier mobility, broad absorption region, and miscibility with fullerene derivatives. In this regard, the HOMO level of the designed donor materials should been reduced to increase the open circuit voltage (*V*_oc_), because the HOMO of donor and the LUMO of acceptor are closely relate to the *V*_oc_. With the aim to harvest more sunlight, the energy gaps of the designed donor materials should been decreased, which results in an increase in the short circuit current density (*J*_sc_) (Loser et al., 2017; Maglione et al., 2017; Zhang et al., 2017). Moreover, a key factor that impact on the efficient exciton splitting and charge dissociation is the downhill energetic driving force (Δ*E*_L−L_), which is the energy differences between the LUMOs of the donor and acceptor. The Δ*E*_L−L_ value should be about 0.3 eV to ensure efficient charge transfer, exciton splitting, and charge dissociation (Scharber et al., 2006). Therefore, an ideal donor material should have narrowing the HOMO-LUMO gap (*E*_g_) and suitable FMOs energy levels with PCBM ([6,6]-phenyl-C61-butyric acid methyl ester) and PC71BM ([6,6]-phenyl-C71 butyric acid methyl ester), which are widely employed as acceptors in OSCs (He et al., 2007; Lenes et al., 2008). Among the various approaches to design organic π-conjugated SMs materials with the long range absorption, one of the successful approaches is to incorporate the electron-donating (D) and electron-accepting (A) moieties in π-conjugated SMs (Qu and Tian, 2012; Guo et al., 2017; Wang et al., 2017). The FMOs energy levels, absorption and emission properties as well as intermolecular charge transfer of these materials can be tuned effectively by altering the chemistries of the donor and acceptor units. At the same time, adjusting the donor and acceptor units can also affect their self-assembly in the solid state. Among the various D–A type SMs donors for OSCs, diketopyrrolo[3,4-c]pyrrole (DPP)-based molecules are promising building blocks owing to their excellent coplanarity, broader absorption region, and thermal stability (Chen et al., 2013; Lin et al., 2013; Zhang et al., 2014). Furthermore, the introduction of the planar heteroarenes into the strong electron-withdrawing DPP-based molecules backbones can lead to lower the band gap because of increasing effective conjugation length (Dutta et al., 2012; Patra et al., 2013). In addition, star-shaped SMs materials with π-conjugated arms can harvest sunlight effectively because of their extended dimensionality. Meanwhile, their steric hindrances can prevent the formation of an ordered, long-range, and coplanar π-π stacking, which are beneficial for their charge transport property (Irfan et al., 2017). Therefore, the star-shaped D–A type DPP-based molecules may possess narrower band gap, broader absorption region, strong light absorption, and high charge carrier mobility (Sharma et al., 2014; Shiau et al., 2015).

**Scheme 1:**
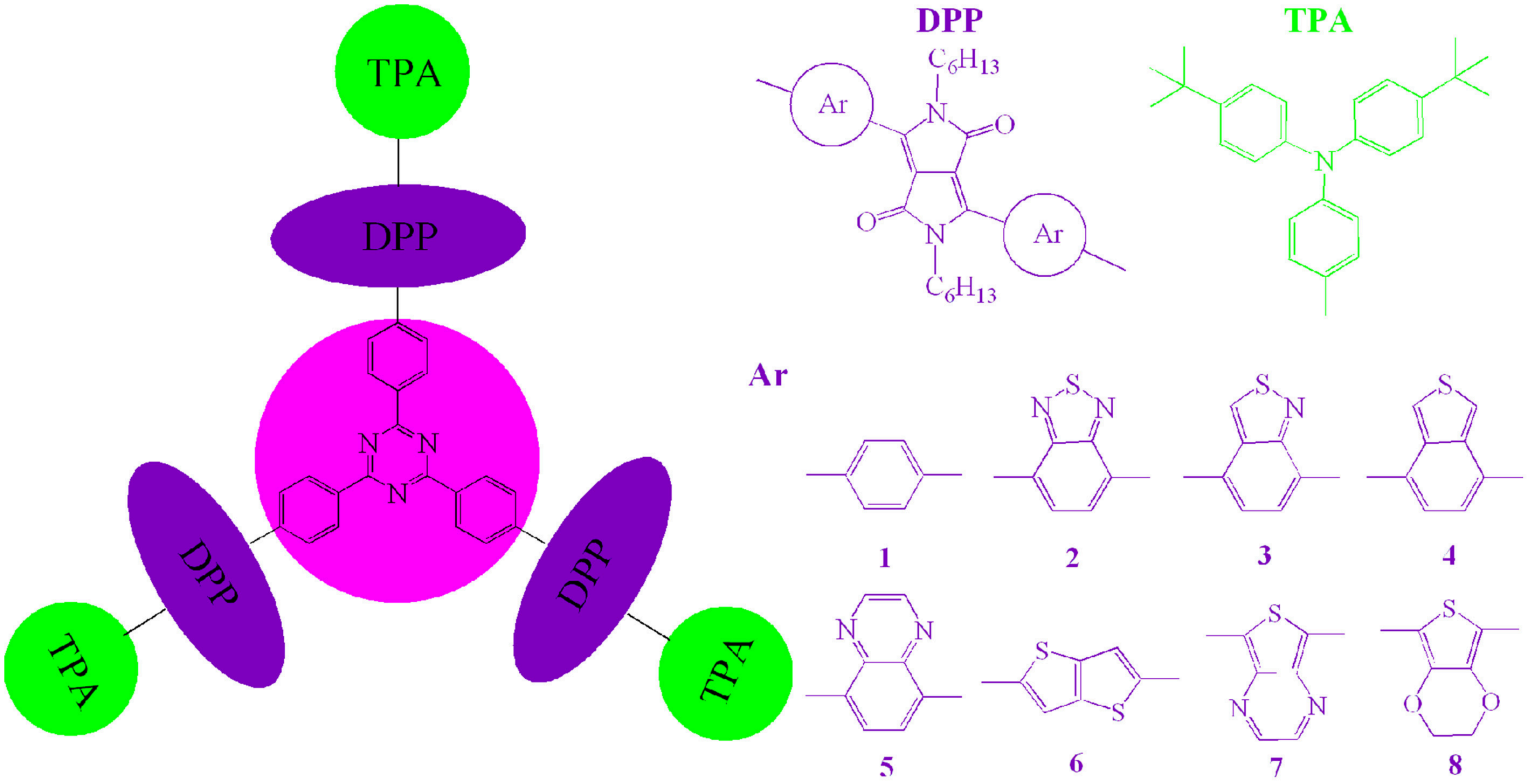
Molecule models of **1**–**8** investigated in this work.

Considering these merits and characteristics mentioned above, in this contribution, we report the design of a series of novel star-shaped DPP-based molecules with electron-accepting 2,4,6-triphenyl-1,3,5-triazine (TPTA) as core, electron accepting DPP derivatives as arms, and electron-donating triphenylamine derivatives (TPA) as end groups for OSCs applications (as shown in Scheme 1). With the aim to investigate the relationships between structure and properties of the designed molecules, the different planar heteroarenes have been introduced into the side of DPP molecules backbones in the star-shaped molecules. The HOMO energy (*E*_HOMO_), LUMO energy (*E*_LUMO_), HOMO–LUMO gap (*E*_g_), energetic driving force Δ*E*_L−L_, and absorption spectra of the designed molecules were systematically investigated by applying density functional theory (DFT) and time-dependent DFT (TD-DFT) methodology. The charge transfer properties (reorganization energy, λ) were also simulated.

## Computational Details

**Figure 1:**
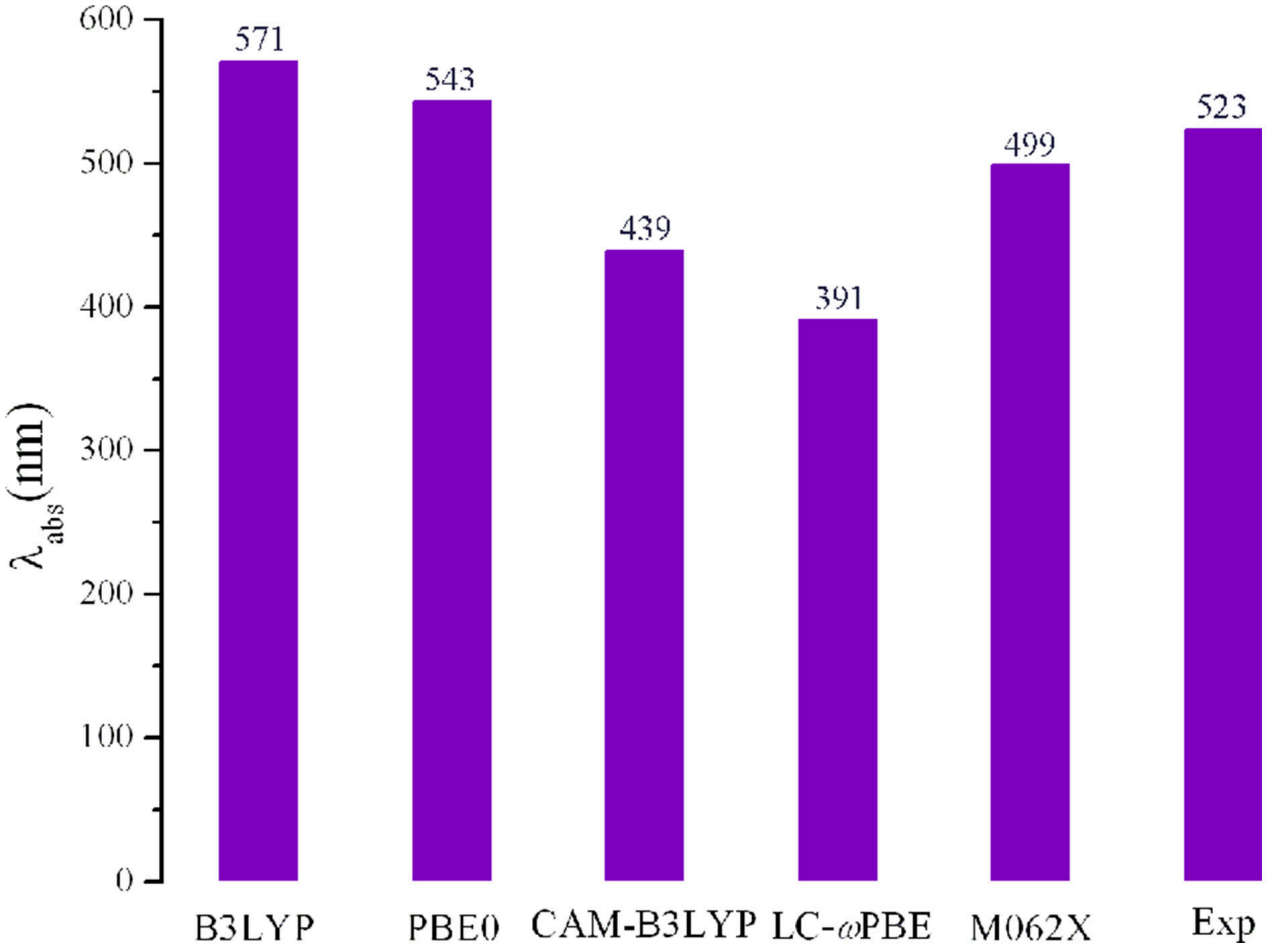
Calculated absorption wavelengths (λ_abs_) of **1** using various functionals, together with the experimental result.

Using the Gaussian 09 W software package (Frisch et al., 2009), all the geometry optimizations and frequency for the designed molecules in the gas phase were performed with the DFT method. No imaginary frequency was used to ensure the nature of the stationary point for the optimized molecules. On the basis of the optimized structures, the absorption spectra of the designed molecules were predicted using the TD-DFT method. The 6-31G (d,p) basis set was employed for all calculations in this work. For the FMOs energy levels of the designed molecules, because it is difficult to describe the virtual orbitals theoretically (Wu et al., 2013). The LUMO energy levels can be calculated with the equation, *E*_LUMO_ = *E*_HOMO_ + *E*^ex^, where *E*^ex^ represents the first vertical excited energy (Zhang and Musgrave, 2007; Ku et al., 2011; Zhang et al., 2012). A crucial step in the theoretical investigations is to select an appropriate exchange correlation functional. With the aim to select an appropriate approach, we chose various functionals such as B3LYP (Lee et al., 1988), PBE0 (Adamo and Barone, 1999), LC-wPBE (Tawada et al., 2004), M062X (Zhao and Truhlar, 2008), and CAM-B3LYP (Yanai et al., 2004) to optimize the geometries of the parent molecule **1**. Based on the optimized geometries, the absorptions were predicted using the TD-DFT method. The longest wavelengths of absorption (λ_abs_) as well as the experimental data are shown in Figure 1. As showing Figure 1, the calculated λ_abs_ value obtained at PBE0 (543 nm) level provided better agreement with the experimental value (523 nm) (Shiau et al., 2015) than those obtained with other levels of theory, with the deviation being 20 nm. Although B3LYP appeared adapted to 1,3,5-triazine and DPP derivatives in literature (Feng et al., 2014; Vala et al., 2014; Jin, 2015; Jin and Irfan, 2015; Jin and Xiao, 2015; Fujii et al., 2016), the λ_abs_ value obtained at the B3LYP/6-31G (d,p) level is worsen accordance with the experimental data (the deviation is 48 nm) than that for at the PBE0/6-31G (d,p) level (the deviation is 20 nm). Additionally, we also calculated the FMOs energy levels of molecule **1** using both at PBE0 and B3LYP methods. The calculated *E*_HOMO_ and *E*_LUMO_ values (−5.04 and −2.76 eV) at the PBE0/6-31G (d,p) level are more close to the electrochemical measurements data (−5.47 and −3.41 eV) (Shiau et al., 2015) than those obtained at the B3LYP/6-31G (d,p) level (−4.82 and −2.67 eV), respectively. Furthermore, in order to make further investigation of the validity of the selected approach, both PBE0 and B3LYP methods were also employed to optimize the structure of PCBM and PC71BM. The calculated *E*_HOMO_ and *E*_LUMO_ of PCBM and PC71BM along with available experimental data are listed in Table S1. Inspection of Table S1 reveals clearly that the *E*_HOMO_ and *E*_LUMO_ at the PBE0/6-31G (d,p) level of PCBM are −5.98 and −3.99 eV, and the corresponding values of PC71BM are −5.92 and −3.82 eV, respectively. These are well reproduce the experimental values of PCBM (−6.00 and −3.80 eV) (Jeon et al., 2016) and PC71BM (−6.00 and −3.95 eV) (Chandrasekharam et al., 2014), respectively. However, at B3LYP/6-31G (d,p) level, the calculated *E*_HOMO_ and *E*_LUMO_ of PCBM are −5.67 and −3.75 eV, while the corresponding values of PC71BM are −5.61 and −3.60 eV, respectively. It was noticed that B3LYP overestimate the *E*_HOMO_ and *E*_LUMO_ of PCBM and PC71BM. The B3LYP overestimate the *E*_HOMO_ and *E*_LUMO_ compared with experimental value, as reported in the literature (Blouin et al., 2008; Xiao et al., 2010; Abbotto et al., 2012). Therefore, PBE0 functional is reasonable to investigate the current system. In order to obtain insight into the method to describe and the influence of functionals on the optical properties, the absorption spectrum of the designed molecules were also simulated at B3LYP/6-31G (d,p) levels.

It is well-known that the reorganization energy (λ) play the dominant role in the effective charge transfer according to the Marcus theory (Marcus, 1964, 1993). A good charge transfer materials should possess lower λ values, which led to higher charge transfer rate. We only pay attention to the internal reorganization energy in this work owing to the low dielectric constant of medium in OSCs materials (Marcus, 1964). The electron (λ_e_) and hole (λ_h_) reorganization energy can be expressed as follows (Köse et al., 2007; Sancho-García, 2007):

(1) $$\begin{array}{l} \lambda e=\left(E_{0}^{-}-E_{-}^{-}\right)+\left(E_{-}^{0}-E_{0}^{0}\right) \end{array}$$

(2) $$\begin{array}{l} \lambda h=\left(E_{0}^{+}-E_{+}^{+}\right)+\left(E_{+}^{0}-E_{0}^{0}\right) \end{array}$$

Here, $E_{0}^{\pm }$ and $E_{\pm }^{\pm }$ are the energies of the cationic (anionic) states with the optimized neutral and cationic (anionic) geometry, respectively. $E_{\pm }^{0}$ and $E_{0}^{0}$ represent the energy of the neutral states with the optimized geometry of the cationic (anionic) and neutral structures, respectively. The λ_e_ and λ_h_ of the designed molecules were predicted at the PBE0/6-31G(d,p) level.

It is noteworthy that the stability is the most important criteria to evaluate the nature of devices for OSCs. Generally, the absolute hardness (η) was applied to explore the stability of the materials. From a viewpoint of conceptual density functional theory, the η values of the designed molecules were calculated with the following equation (Cheung and Troisi, 2010):

(3) $$\begin{array}{l} \eta =\frac{1}{2}\left(\frac{\partial \mu }{\partial N}\right)=\frac{1}{2}\left(\frac{\partial^{2}E}{\partial N^{2}}\right)=\frac{AIP-AEA}{2} \end{array}$$

Here, *μ* and *N* correspond to the chemical potential and total electron number, respectively. The adiabatic ionization potential (*AIP*) is the energy difference between the cation radical specie and its neutral specie, while the adiabatic electron affinity (*AEA*) represents the energy difference between the neutral molecule and its anion radical molecule.

## Results and Discussion

### Frontier Molecular Orbitals and Band Gaps

**Figure 2:**
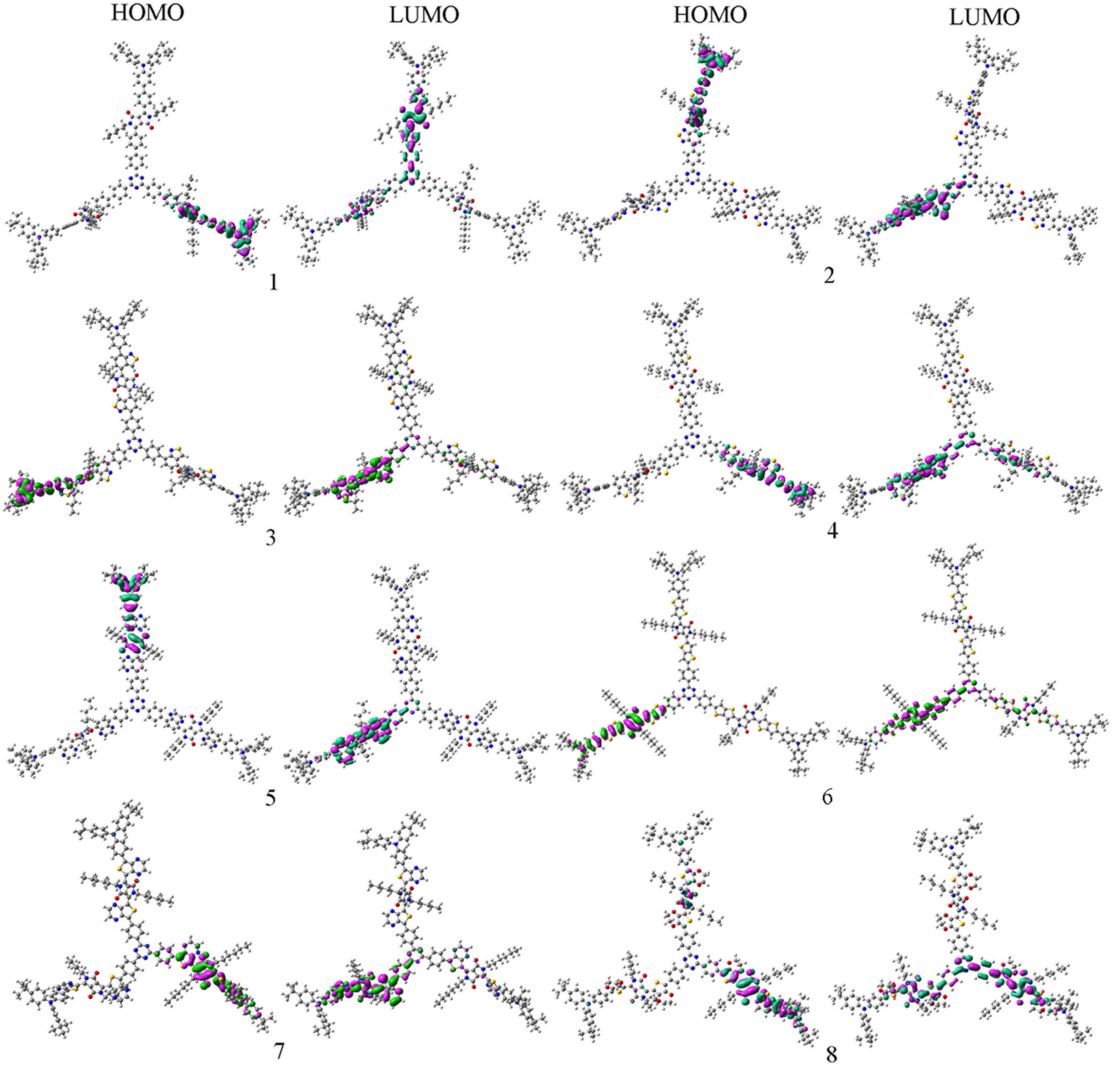
The FMOs of the designed molecules at the PBE0/6-31G(d,p) level.

In order to characterize the optical and electronic properties, we investigated the distributions of the FMOs for the designed molecules. The distribution of HOMOs and LUMOs are plotted in Figure 2. Based on Mulliken population analysis, molecular orbital contribution (%) from core TPTA, arms DPP, and end groups TPA to the FMOs of **1**–**8** are given in Table 1. The corresponding contributions (%) from TPTA, DPP, and TPA groups to the HOMOs-1 and LUMOs+1 of **1**–**8** are given in Table S2. As visualized in Figure 2, the distribution of HOMOs and LUMOs are spread over the conjugated backbone and show π orbital features. The HOMOs are mainly localized on the arm groups DPP and end groups TPA with only minor contributions from the core fragments TPTA. The sum contributions of DPP and TPA fragments are larger than 96.1%, while the corresponding values of TPTA fragments are within 3.9% for HOMOs. On the contrary, the LUMOs are mainly distributed on the DPP and TPTA moieties with minor contributions from TPA fragments. The sum contributions of DPP and TPTA fragments for LUMOs are larger than 94.4%, while the corresponding values of TPA fragments are within 5.6%. Obviously, the contributions of both DPP and TPTA fragments for LUMOs are larger than those of for HOMOs, respectively. The contributions of TPA fragments to LUMOs are decreased compared with those of to HOMOs, respectively. Similar phenomena are found for the HOMOs-1 and LUMOs+1 of **1**–**8**. The changes in contributions suggest that the electronic density flow from the end groups TPA to the arms groups DPP and cores groups TPTA for HOMOs → LUMOs excitations. This indicates that the end groups TPA serve as donors, whereas, the arm groups DPP and core groups TPTA serve as acceptors, respectively.

**Table 1:** Molecular orbital contribution (%) from core TPTA, arms DPP, and end groups TPA to the FMOs of **1**–**8** at the PBE0/6-31G(d,p).

**Species**	**HOMO**			**LUMO**		
	**TPTA**	**DPP**	**TPA**	**TPTA**	**DPP**	**TPA**
1	0.7	32.5	66.4	17.8	79.4	2.8
2	0.6	34.5	64.9	8.3	88.4	3.3
3	0.3	18.3	81.4	8.4	88.5	3.1
4	0.8	53.7	45.5	17.2	80.9	2.0
5	0.3	29.8	69.9	8.4	89.	2.5
6	2.4	76.5	21.1	16.9	79.9	3.2
7	3.9	71.1	25.0	11.6	82.7	5.6
8	3.1	69.6	27.3	25.8	69.9	4.3

*TPTA, 2,4,6-triphenyl-1,3,5-triazine moieties; DPP, diketopyrrolopyrrole moieties; TPA, triphenylamine moieties*.

**Figure 3:**
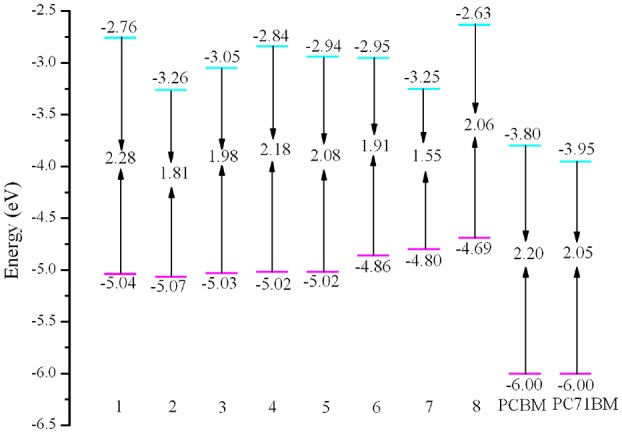
Evaluation of calculated FMO energies for the designed molecules as well as FMO energies for PCBM and PC70BM at the PBE0/6-31G(d,p) level.

It is worth noting that the *E*_HOMO_, *E*_LUMO_, *E*_g_, and Δ*E*_L−L_ are strongly related to the optical and electronic properties. The calculated values of *E*_HOMO_, *E*_LUMO_, *E*_g_, and Δ*E*_L−L_ of the designed molecules are given in Table 2 and depicted in Figure 3. As shown in Figure 3, it is clear that the *E*_HOMO_ values of **3**–**8** increase, while the corresponding value of **2** decreases compared with that of **1**. The *E*_HOMO_ values is in the order of **8** > **7** > **6** > **4** ≈ **5** > **3** > **1** > **2**. On the other hand, the *E*_LUMO_ values of **2**–**7** decrease, while the corresponding value of **8** increases compared with that of **1**. The sequence of *E*_LUMO_ values is **8** > **1** > **4** > **5** > **6** > **3** > **2** > **7**. Therefore, the *E*_g_ values of **2**–**8** decrease compared with that of **1**. The *E*_g_ values are in the order of **1** < **4** < **5** < **8** < **3** < **6** < **2** < **7**. The analysis indicates that the decrease of *E*_g_ is mainly attributable to the increased *E*_HOMO_ and declined *E*_LUMO_. The reducing the *E*_g_ of the designed molecules should leads to bathochromic shifts of the maximum absorption compared with that of **1**. Consequently, the introduction of different groups to the side of DPP molecules backbones in the star-shaped molecules can tune the FMOs energy and *E*_g_ values of the original molecule. It provides a powerful strategy for design high-performance and desirable donor novel SMs. Furthermore, in order to ensure efficient charge transfer, the Δ*E*_L−L_ values must exceed the binding energy (0.2 ~ 1.0 eV) (Hill et al., 2000; Knupfer, 2003). From Table 2, it is noteworthy that the Δ*E*_L−L_ values of the designed molecules are all beyond the binding energy with regard to PCBM and PC71BM as acceptors. It is clear that the sequence of the values of Δ*E*_L−L_ with regard to PCBM and PC71BM are all **8** > **1** > **4** > **5** > **6** > **3** > **7** > **2**. In addition, the differences between the *E*_HOMO_ of **1**–**8** and the *E*_LUMO_ of PCBM and PC71BM are larger than 0.73 and 0.56 eV, respectively. Thus, it is quite clear that the designed molecules can provide match well with PCBM and PC71BM as acceptors.

**Table 2:** Calculated *E*_HOMO_, *E*_LUMO_, *E*_g_, and Δ*E*_L−L_ (all in eV) for investigated molecules at the PBE0/6-31G(d,p).

**Species**	***E*_**HOMO**_**	***E*_**LUMO**_**	***E*_**g**_**	**Δ*E*_L-L_^a^**	**Δ*E*_**L-L**_^b^**
1	−5.04	−2.76	2.28	1.23	1.06
2	−5.07	−3.26	1.81	0.73	0.56
3	−5.03	−3.05	1.98	0.94	0.77
4	−5.02	−2.84	2.18	1.15	0.98
5	−5.02	−2.94	2.08	1.05	0.88
6	−4.86	−2.95	1.91	1.04	0.87
7	−4.80	−3.25	1.55	0.74	0.57
8	−4.69	−2.63	2.06	1.36	1.19

a *Energetic driving force for PCBM as donor*.

b *Energetic driving force for PC71BM as donor*.

### Absorption Spectra

**Figure 4:**
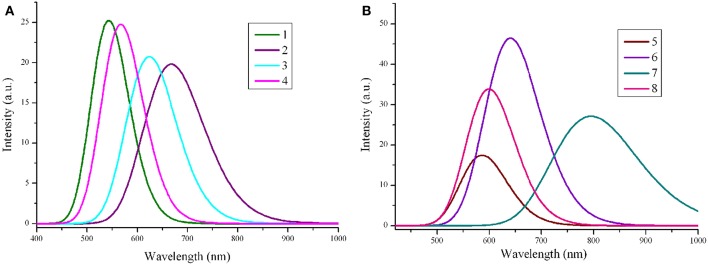
The calculated absorption spectra of the investigated molecules (value of full width at half maximum is 3,000 cm^−1^). **(A)** Molecules **1**–**4**; **(B)** Molecules **5**–**8**.

The absorption wavelengths λ_abs_ (in nm), the oscillator strength *f* , and main assignments (coefficient), and the absorption region *R* of **1**–**8** at the PBE0/6-31G(d,p) level are listed in Table 3. *R* denotes for the difference of the longest and shortest wavelength values with oscillator strength larger than 0.01 considering the first 15 excited states (see Table S3). The simulated absorption spectra of **1**–**8** are shown in Figure 4, which were plotted by using the GaussSum 1.0 program (O'Boyle and Vos, 2003). As expected, the results displayed in Table 3 reveals that the λ_abs_ of **2**–**8** exhibit bathochromic shifts compared with that of **1**. The bathochromic shifts values of **2**–**8** are 143, 83, 26.1, 53.8, 104.6, 256.2, and 59.0 nm (3834, 2439, 844, 1658, 2971, 5897, and 1803 cm^−1^), respectively. Moreover, the λ_abs_ values are in the order of **7** > **2** > **6** > **3** > **8** > **5** > **4** > **1**, which is in excellent agreement with the corresponding reverse sequence of their *E*_g_ values. It reveals that the introduction of different groups to the side of DPP molecules backbones leads to bathochromic shifts of the maximum absorption for the original molecule. The order of the bathochromic shifts values compared with that of **1** is thieno[3,4-b]pyrazine (**7**) > benzo[c][1,2,5]thiadiazole (**2**) > thieno[3,2-b]thiophene (**6**) > benzo[c]isothiazole (**3**) > 2,3-dihydrothieno[3,4-b][1,4]dioxine (**8**) > quinoxaline (**5**) > benzo[c]thiophene (**4**). Additionally, one can find that **6**–**8** have larger oscillator strengths, while the corresponding values of **2**, **3**, and **5** possess slightly < that of **1**. The oscillator strength value of **4** is almost equal to that of **1**, indicating that the designed molecules shown large absorption intensity. At the same time, the designed molecules have large absorption region *R* (82.3–225.7 nm). The *R* values of 2–8 are larger than that of parent compound 1. It suggests that the introduction of different groups to the side of DPP molecules backbones lead to the increase of R values compared with parent molecule 1. The order of *R* values compared with that of **1** is thieno[3,4-b]pyrazine (**7**) > benzo[c]isothiazole (**3**) > 2,3-dihydrothieno[3,4-b][1,4]dioxine (**8**) > benzo[c][1,2,5]thiadiazole (**2**) > thieno[3,2-b]thiophene (**6**) > quinoxaline (**5**) > benzo[c]thiophene (**4**). It is noticeable that a good overlap between the absorption spectrum of the designed molecules and the solar emission spectrum, which can improve the light-absorption efficiency. It clearly shows that the introduction of different groups can extend the absorption spectrum toward longer wavelengths, which is beneficial to harvest more sunlight. These results imply that the designed compounds have strong absorption and are expected to be the promising candidates for donor materials in OSCs applications.

**Table 3:** The electronic transition, absorption wavelengths λ_abs_ (in nm), the oscillator strength *f* , main assignments (coefficient), and the absorption region *R* of **1**–**8** at the TD-PBE0/6-31G(d,p)//PBE0/6-31G(d,p) level, along with available experimental data.

**Species**	**λ_abs_**	***f***	**Assignment**	***R***
1	543.4	1.79	H → L (0.12) H-2 → L (0.50) H-1 → L (0.20)	72.2
2	686.4	1.30	H-2 → L (0.66) H-5 → L (−0.17)	142.2
3	626.4	1.37	H → L (0.59) H → L+2 (0.21) H-2 → L+1 (0.21)	155.3
4	569.5	1.79	H → L (0.30) H-1 → L (0.43) H-1 → L+2 (−0.22)	82.3
5	597.2	1.01	H-2 → L (0.64) H-5 → L (0.23)	106.1
6	648.0	3.13	H → L (0.60) H → L+2 (0.21) H-2 → L+1 (−0.19)	139.1
7	799.6	2.19	H → L (0.24) H-2 → L (0.45) H → L+1 (−0.41)	225.7
8	602.4	2.83	H → L (0.51) H-2 → L (0.20), H-2 → L+1 (0.30)	152.7
Exp	523			

*Exp, Experimental results of ***1***in thin film were taken from Sharma et al. (2014), Shiau et al. (2015)*.

The calculated λ_abs_, *f* , and main assignments (coefficient) of **1**–**8** at the B3LYP/6-31G(d,p) level are listed in Table S4. Comparing the results shown in Table 3 with Table S4, one can find that the calculated λ_abs_ values of **1**–**8** at the B3LYP/6-31G(d,p) are larger than those obtained at the PBE0/6-31G(d,p), respectively. The differences between λ_abs_ at the B3LYP/6-31G(d,p) and PBE0/6-31G(d,p) levels are about 30 ~ 50 nm. It should be mentioned that although the *E*_HOMO_ and *E*_LUMO_ are overestimated with both the PBE0 and B3LYP functionals, the predicted λ_abs_ values using PBE0 are found to be closer to the experimental findings. The trend for λ_abs_ at B3LYP/6-31G(d,p) is similar to using PBE0/6-31G(d,p) method. Obviously, B3LYP functional underestimate the *E*_g_ value, resulting in the large λ_abs_ compared with experimental value. Considering the FMOs energy levels and the predicted absorption spectra mentioned above, the PBE0/6-31G(d,p) approach is the best choice to well reproduce the experimental results. Therefore, the λ, η, *AIP*, and *AEA* of the designed molecules were computed at PBE0/6-31G(d,p).

### Adiabatic Ionization Potential and Electron Affinity

It is well known that *AIP* and *AEA* are two major parameters that determine the charge transfer behavior for materials. The carrier polarity of materials can be adjusted by the *AIP* and *AEA* values (Chen and Chao, 2005; Liu et al., 2010). The lower *AIP* and higher *AEA* revealed that material would be better hole and electron transporter, respectively (Li et al., 2012). The calculated *AIP* and *AEA* of **1**–**8** are collected in Table 4. Obviously, the results displayed in Table 4 show that the increasing sequence of *AIP* values is **8** < **7** < **6** < **4** < **5** < **1** < **3** < **2**. On the other hand, the decreasing order of *AEA* values is **7** > **2** > **3** > **6** > **5** > **4** > **1** > **8**. It indicates that the introduction of benzo[c]thiophene (**4**), quinoxaline (**5**), thieno[3,2-b]thiophene (**6**), and thieno[3,4-b]pyrazine (**7**) groups can decrease/increase *AIP*/*AEA* values compared with that of **1**. However, the benzo[c][1,2,5]thiadiazole (**2**) and benzo[c]isothiazole (**3**) groups can increase both *AIP* and *AEA* values, whereas 2,3-dihydrothieno[3,4-b][1,4]dioxine (**8**) group can decrease both *AIP* and *AEA* values compared with that of **1**. It is noticeable that the introduction of different aromatic heterocyclic group to the side of DPP molecules backbones can affect the *AIP* and *AEA* of the designed molecules.

**Table 4:** Calculated molecular *AIP* and *AEA* (both in eV) of **1**–**8** at the PBE0/6-31G(d,p) level.

**Species**	***AIP***	***AEA***
1	5.404	1.953
2	5.435	2.372
3	5.406	2.272
4	5.362	1.979
5	5.378	2.068
6	5.209	2.196
7	5.163	2.449
8	5.048	1.781

### Reorganization Energies and Stability Properties

The calculated λ_e_, λ_h_, and η of **1**–**8** are listed in Table 5. It is worth noting that the lower the reorganization energy values can be beneficial to the higher charge transfer rate (Marcus, 1964, 1993). Usually, tris(8-hydroxyquinolinato)aluminum(III) (Alq3, λ_e_ = 0.276 eV) and N,N′-diphenyl-N,N′-bis(3- methlphenyl)-(1,1′-biphenyl)-4,4′-diamine (TPD, λ_h_ = 0.290 eV) are taken as typical electron and hole transport materials, respectively (Gruhn et al., 2002; Lin et al., 2005). It is clear from Table 5 that the λ_h_ values of **1**–**8** (0.046–0.129 eV) are smaller than that of TPD. It indicates that the hole transfer rates of **1**–**8** are higher than that of TPD. On the other hand, the λ_e_ values of **1**–**8** (0.091–0.209 eV) are smaller than that of Alq3. It implies that the electron transfer rates of **1**–**8** might be higher than that of Alq3. The λ_h_ values of **1**–**5** are slightly smaller than those of **6**–**8**, suggesting that the hole transfer rates of **1**–**5** should be higher than those of **6**–**8**, respectively. It indicates that the introduction of benzene (**1**), benzo[c][1,2,5]thiadiazole (**2**), benzo[c]isothiazole (**3**), benzo[c]thiophene (**4**), and quinoxaline (**5**) groups may lead to higher charge transfer rates than that of thieno[3,2-b]thiophene (**6**), thieno[3,4-b]pyrazine (**7**), and 2,3-dihydrothieno[3,4-b][1,4]dioxine (**8**) groups, respectively. The λ_e_ values is in the order of **3** > **5** > **4** > **1** > **8** > **7** > **2** > **6**. It suggests that molecules with benzo[c][1,2,5]thiadiazole (**2**), thieno[3,2-b]thiophene (**6**), thieno[3,4-b]pyrazine (**7**), and 2,3-dihydrothieno[3,4-b][1,4]dioxine (**8**) possess higher electron transfer rates, while molecules with benzo[c]isothiazole (**3**), benzo[c]thiophene (**4**), and quinoxaline (**5**) groups have lower electron transfer rates compared with that of **1**, respectively. Additionally, the λ_h_ values of **1**–**5** are smaller than those of their λ_e_ values, suggesting that the carrier mobility of the hole is larger than that of the electron. However, the λ_e_ values of **6** and **7** are smaller than those of their λ_h_ values, implying that the carrier mobility of the electron is larger than that of the hole. Moreover, the differences between λ_e_ and λ_h_ values of the designed molecules are in the region of 0.00 ~ 0.087 eV except the corresponding value of **3** is 0.163 eV, respectively. It indicates that they exhibit better equilibrium feature for hole and electron transport. Therefore, **1**, **2**, and **4**–**8** are expected to be the promising candidates for ambipolar charge transports materials, whereas **3** can be used as hole and electron transport material.

**Table 5:** Calculated molecular λ_e_, λ_h_, and η (all in eV) of **1**–**8** at the PBE0/6-31G(d,p) level.

**Species**	**λ_**h**_**	**λ_**e**_**	**η**
1	0.054	0.134	1.725
2	0.058	0.112	1.532
3	0.046	0.209	1.567
4	0.090	0.135	1.692
5	0.051	0.138	1.655
6	0.107	0.091	1.506
7	0.129	0.122	1.357
8	0.128	0.128	1.633

The absolute hardness η of **1**–**8** were calculated and shown in Table 5. The η values is in the order of **1** > **4** > **5** > **8** > **3** > **2** > **6** > **7**. Inspection of Table 5 reveals clearly that the η values of **2**–**8** are smaller slightly than the value of **1**, which may be owing to the steric hindrances of the heterocyclic groups introduced to the side of DPP backbones in star-shaped DPP-based molecules. It implies that the introduction of different heterocyclic groups do not significantly affect the stability of the designed molecules.

## Conclusion

In this contribution, a series of novel star-shaped molecules have been systematically investigated. Their electronic, optical, and charge transport properties studied using DFT and TD-DFT approaches. The calculated results show that the introduction of different groups to the side of DPP backbones in the star-shaped molecules can tune the FMOs energy and *E*_g_ values of the original molecule. The designed molecules can provide match well with PCBM and PC71BM as acceptors. Additionally, the λ_abs_ of **2**–**8** show bathochromic shifts compared with that of the original molecule **1**, respectively. The introduction of different groups can extend the absorption spectrum toward longer wavelengths, which is beneficial to harvest more sunlight. Our results suggest that the designed molecules are expected to be the promising candidates for ambipolar charge transport materials except molecule with benzo[c]isothiazole group (**3)** can be used as hole and electron transport material. Moreover, the different substituent groups do not significantly affect the stability of the designed molecules.

## Author Contributions

RJ conceived and designed the research and headed, wrote, and revised the manuscript. XZ contributed to the performance and analysis of the frontier molecular orbitals, absorption spectra the reorganization energies. Both authors contributed to manuscript revision, read, and approved the submitted version.

### Conflict of Interest Statement

The authors declare that the research was conducted in the absence of any commercial or financial relationships that could be construed as a potential conflict of interest.
