# Supplementary material for: Rational Design of Low-Band Gap Star-Shaped Molecules With 2,4,6-Triphenyl-1,3,5-triazine as Core and Diketopyrrolopyrrole Derivatives as Arms for Organic Solar Cells Applications
Source: Front Chem. 2019 Mar 19;7:122. doi: 10.3389/fchem.2019.00122 (PMC6433785; doi:10.3389/fchem.2019.00122)
Supplement: Supplementary file 1 [file Table_1.DOC]

**Rational design of low-band gap star-shaped molecules with 2,4,6-triphenyl-1,3,5-triazine as core and diketopyrrolopyrrole derivatives as arms for organic solar cells applications**

Xinhao Zhang, Ruifa Jin*

*Inner Mongolia Key Laboratory of Photoelectric Functional Materials, College of Chemistry and Chemical Engineering, Chifeng University, Chifeng 024000, China*

**Supplementary**

**List of Contents**

**Table S1** The *E*_HOMO_ and *E*_LUMO_ (in eV) of PCBM and PC71BM at PBE0/6-31G (d,p) and B3LYP/6-31G (d,p) levels, along with available experimental data.

**Table S2** Molecular orbital contribution (%) from core TPTA, arms DPP, and end groups TPA to the HOMO-1 and LUMO+1 of **1**–**8** at the PBE0/6-31G(d,p) level.

**Table S3** The *λ*_abs_ and corresponding oscillator strength *f* (in parenthesis) and absorption region *R* of the first fifteen excited states for **1**–**8** obtained by the TD-PBE0/6-31G(d,p) level.

**Table S4** The electronic transition, absorption wavelengths λ_abs_ (in nm), the oscillator strength *f*, and main assignments (coefficient) of **1**–**8** at the TD-B3LYP/6-31G(d,p)//B3LYP/6-31G(d,p) level, along with available experimental data.

**Table S1**

**The *E*_HOMO_ and *E*_LUMO_ (in eV) of PCBM and PC71BM at PBE0/6-31G (d,p) and B3LYP/6-31G (d,p) levels, along with available experimental data**

| Methods | PCBM | |  | PC71BM | |
| --- | --- | --- | --- | --- | --- |
|  | *E*_HOMO_ | *E*_LUMO_ |  | *E*_HOMO_ | *E*_LUMO_ |
| PBE0/6-31G (d,p) | -5.98 | -3.99 |  | -5.92 | -3.82 |
| B3LYP/6-31G (d,p) | -5.67 | -3.75 |  | -5.61 | -3.60 |
| EXP | -6.00^a^ | -3.80^a^ |  | -6.00^b^ | -3.95^b^ |

^a^ Jeon, I., Delacou, C., Nakagawa,T., and Yutaka Matsuo, Y. (2016) Chem. Asian. J. 11, 1268−1272. ^b^ Chandrasekharam, M., Anil Reddy, M., Ganesh, K., Sharma, G. D. Singh, S. P., J. and Rao, L. (2014) Org. Electron. 15 2116–2125.

**Table S2**

Molecular orbital contribution (%) from core TPTA, arms DPP, and end groups TPA to the HOMO-1 and LUMO+1 of **1**–**8** at the PBE0/6-31G(d,p).

| Species | HOMO-1 | | |  | LUMO+1 | | |
| --- | --- | --- | --- | --- | --- | --- | --- |
|  | TPTA^a^ | DPP^b^ | TPA^c^ |  | TPTA | DPP | TPA |
| **1** | 0.7 | 33.6 | 66.4 |  | 18.9 | 78.4 | 2.7 |
| **2** | 0.6 | 34.0 | 65.4 |  | 11.3 | 86.1 | 2.6 |
| **3** | 0.3 | 18.3 | 81.4 |  | 8.5 | 88.3 | 3.2 |
| **4** | 0.7 | 47.0 | 52.2 |  | 17.4 | 80.5 | 2.1 |
| **5** | 0.4 | 31.1 | 68.5 |  | 12.1 | 85.6 | 2.2 |
| **6** | 2.1 | 71.8 | 26.1 |  | 17.0 | 79.9 | 3.0 |
| **7** | 3.5 | 70.5 | 26.0 |  | 12.8 | 81.4 | 5.8 |
| **8** | 2.9 | 68.4 | 28.7 |  | 25.0 | 70.6 | 4.3 |

^a^TPTA: 2,4,6-triphenyl-1,3,5-triazine moieties; ^b^ DPP: diketopyrrolopyrrole moieties; ^c^ TPA:

**Table S3** The *λ*_abs_ and corresponding oscillator strength *f* (in parenthesis) and absorption region *R* of the first fifteen excited states for **1**–**8** obtained by the TD-PBE0/6-31G(d,p) level.

| Species | **1** | **2** | **3** | **4** | **5** | **6** | **7** | **8** |
| --- | --- | --- | --- | --- | --- | --- | --- | --- |
| S_1_ | 543.4(1.80) | 686.4(1.30) | 626.4(1.37) | 569.5(1.79) | 597.2(1.02) | 648.0(3.14) | 799.6(2.19) | 602.4(2.84) |
| S_2_ | 542.6(1.59) | 655.8(1.08) | 621.7(1.30) | 565.1(1.39) | 579.3(1.15) | 634.0(3.16) | 792.9(1.40) | 597.0(1.59) |
| S_3_ | 533.3(0.11) | 644.1(0.49) | 613.7(0.20) | 554.1(0.25) | 573.2(0.30) | 610.3(0.22) | 753.6(0.19) | 571.7(0.33) |
| S_4_ | 495.5(0.00) | 599.6(0.00) | 555.2(0.00) | 503.7(0.00) | 533.2(0.04) | 588.8(0.00) | 708.1(0.01) | 544.2(0.00) |
| S_5_ | 494.5(0.00) | 597.1(0.00) | 555.0(0.00) | 501.5(0.00) | 525.6(0.00) | 580.4(0.00) | 704.0(0.00) | 540.8(0.00) |
| S_6_ | 493.1(0.00) | 582.6(0.03) | 553.8(0.00) | 500.0(0.00) | 524.6(0.00) | 577.4(0.00) | 682.7(0.01) | 540.7(0.00) |
| S_7_ | 484.8(0.01) | 569.7(0.04) | 547.0(0.00) | 496.1(0.11) | 518.5(0.01) | 572.4(0.04) | 677.1(0.00) | 522.3(0.07) |
| S_8_ | 483.7(0.01) | 568.5(0.01) | 546.7(0.00) | 492.2(0.01) | 517.0(0.02) | 564.1(0.05) | 675.9(0.01) | 518.7(0.04) |
| S_9_ | 482.2(0.00) | 563.6(0.00) | 546.5(0.00) | 491.4(0.02) | 506.3(0.03) | 561.1(0.05) | 659.7(0.01) | 517.3(0.07) |
| S_10_ | 476.4(0.02) | 563.3(0.01) | 523.7(0.47) | 490.1(0.07) | 503.0(0.01) | 514.9(0.01) | 637.5(0.010) | 480.5(0.08) |
| S_11_ | 475.5(0.37) | 562.8(0.01) | 518.9(0.46) | 489.8(0.02) | 501.5(0.00) | 512.3(0.07) | 625.3(0.03) | 476.6(0.04) |
| S_12_ | 471.2(0.12) | 559.9(0.02) | 509.9(0.20) | 486.8(0.08) | 501.1(0.01) | 508.9(0.03) | 624.3(0.02) | 471.4(0.04) |
| S_13_ | 447.5(0.00) | 552.3(0.00) | 484.9(0.07) | 454.6(0.00) | 493.2(0.00) | 485.8(0.0) | 588.0(0.09) | 450.2(0.01) |
| S_14_ | 445.3(0.00) | 545.9(0.04) | 475.7(0.00) | 454.1(0.00) | 491.3(0.03) | 480.9(0.00) | 575.0(0.16) | 450.0(0.12) |
| S_15_ | 444.3(0.00) | 544.2(0.05) | 471.1(0.10) | 453.3(0.00) | 491.1(0.02) | 480.5(0.00) | 573.9(0.04) | 449.7(0.02) |
| R | 72.2 | 142.2 | 155.3 | 82.3 | 106.1 | 139.1 | 225.7 | 152.7 |

**Table S4**

The electronic transition, absorption wavelengths λ_abs_ (in nm), the oscillator strength *f*, and main assignments (coefficient) of **1**–**8** at the TD-B3LYP/6-31G(d,p)//B3LYP/6-31G(d,p) level, along with available experimental data.

| Species | λ_abs_ | *f* | Assignment |
| --- | --- | --- | --- |
| **1** | 575.4 | 1.26 | H → L (0.55)  H-1 → L+1 (-0.25)  H-1 → L+2 (0.21) |
| **2** | 738.3 | 1.11 | H-2 → L (0.68)  H-5 → L (-0.11) |
| **3** | 673.6 | 1.01 | H → L (0.65)  H → L+2 (0.17)  H-2 → L+1 (0.12) |
| **4** | 601.4 | 1.31 | H → L (0.57)  H → L+1 (-0.20)  H → L+2 (0.22) |
| **5** | 631.7 | 0.82 | H-2 → L (0.66)  H-5 → L (0.15) |
| **6** | 689.9 | 2.86 | H → L (0.55)  H → L+1 (0.31)  H-2 → L (0.16) |
| **7** | 852.6 | 1.95 | H → L (-0.26)  H-2 → L (0.51)  H → L+1 (0.35) |
| **8** | 635.6 | 2.44 | H → L (0.48)  H-2 → L (0.26),  H-2 → L+1 (0.29) |
| Exp^a^ | 523 |  |  |

^a^ *Exp, Experimental results of* ***1*** *in thin film were taken from Ref.* (Shiau et al.; 2015; Sharma et al.; 2014)*.*
